# Establishment of gestational diabetes risk prediction model and clinical verification

**DOI:** 10.1007/s40618-023-02249-3

**Published:** 2023-12-12

**Authors:** Z.-R. Niu, L.-W. Bai, Q. Lu

**Affiliations:** 1 Department of Endocrinology, First Hospital of Qinhuangdao, Qinhuangdao, 066000 Hebei China; 2 Department of Obstetrics, Qinhuangdao Hospital for Maternal and Child Health, Qinhuangdao, 066000 Hebei China

**Keywords:** Gestational diabetes mellitus, Predictive model, Body mass index, Uric acid

## Abstract

**Objective:**

The present study aimed to evaluate the risk factors for gestational diabetes mellitus (GDM) and build and validate an early risk prediction model of GDM by comparing the differences in the indicators of the first trimester of pregnancy between pregnant women with GDM and non-gestational diabetes mellitus (NGDM). Thus, this study provided a theoretical basis for early intervention of GDM.

**Methods:**

A total of 6000 pregnant women who underwent a routine prenatal examination in Qinhuangdao Maternal and Child Health Hospital (Qinhuangdao City, Hebei Province, China) from January 2016–2022 were retrospectively selected and randomly divided into a modeling cohort (4200 cases) and validation cohort (1800 cases) at a ratio of 3:7. According to the results of oral glucose tolerance test (OGTT), they were divided into NGDM and GDM groups. The modeling cohort consisted of 2975 NGDM and 1225 GDM cases, while the validation cohort consisted of 1281 NGDM and 519 GDM cases. The differences in general conditions and laboratory indicators between different groups were compared, and logistic regression analysis was further used to establish a risk prediction model for GDM in the first trimester. The receiver operating characteristic curve (ROC) and Hosmer–Lemeshow (HL) tests were used to evaluate the prediction of the model efficacy.

**Results:**

Age, pre-pregnancy body mass index (BMI), glycosylated hemoglobin (HbA1c), blood uric acid (UA), triglyceride (TG), and high-density lipoprotein cholesterol (HDL-C) in the first trimester were independent risk factors for GDM (*P* < 0.05). The model equation was Y = 1/{1 + exp[− (− 18.373 + age × 0.065 + BMI × 0.030 + first-trimester HbA1c × 2.519 + UA × 0.014 + TG × 0.224-HDL-C × 0.635)]}. The area under the ROC curve (AUC) of the model cohort was 0.803 (0.788–0.817), the sensitivity was 72.0%, and the specificity was 73.5%. The AUC of the validation cohort was 0.782 (0.759–0.806), the sensitivity was 68.6%, and the specificity was 73.8%. The *P* values of the HL test in both the training and validation sets were > 0.05, indicating a satisfactory model fit.

**Conclusion:**

Age, pre-pregnancy BMI, HbA1C in early pregnancy, blood UA, TG, and HDL-C are independent risk factors for GDM. The risk prediction model established by combining age, pre-pregnancy BMI, and laboratory indicators in the first trimester can provide a theoretical basis for early screening, monitoring, and intervention of GDM high-risk pregnant women.

## Introduction

Gestational diabetes mellitus (GDM) is one of the common complications of pregnancy, caused by the combined effects of environmental and genetic factors. GDM increases the risk of maternal infection and preeclampsia and also leads to premature birth, fetal malformation, and macrosomia, which significantly increases the risk of type 2 diabetes (T2DM) and metabolic diseases in mothers and offspring [1]. With the rapid development of the social economy and the improvement of living standards, the prevalence of GDM is continuously increasing. According to the data from the International Diabetes Federation (IDF) [2], the global prevalence of GDM was 16.7% in 2021. It has become a significant cause of maternal and child mortality worldwide. Thus, early diagnosis and treatment of GDM are crucial to reducing short- and long-term complications in the mother and child [3].

A previous study [4] found that before the diagnosis, the high-risk group of GDM had a tendency of increased blood sugar, and the high-glycemia environment had adverse effects on the fetus. Therefore, identification of pregnant women with GDM in early pregnancy and targeted intervention could reduce the occurrence of the disease, thereby reducing maternal and fetal complications and improving prognosis. Although some early pregnancy GDM prediction models have been put forth, most have not been widely applied clinically. This study aimed to explore the risk factors of GDM to construct a new and accurate GDM risk prediction model that would improve the specificity and sensitivity of GDM prediction and provide a theoretical basis for early screening, monitoring, and intervention of high-risk patients.

## Materials and methods

General data: A total of 6000 pregnant women who underwent a routine prenatal examination in Qinhuangdao Maternal and Child Health Hospital (Qinhuangdao City, Hebei Province, China) from January 2016–2022 were retrospectively selected and randomly divided into a modeling cohort (4200 cases) and a validation cohort (1800 cases) at a ratio of 3:7. The results of oral glucose tolerance test (OGTT) at 24–28 weeks of pregnancy were recorded, and patients were categorized into non-gestational diabetes mellitus (NGDM) and gestational diabetes mellitus (GDM) groups. The modeling cohort comprised 2975 cases in the NGDM group and 1225 GDM cases, while the validation cohort consisted of 1281 and 519 NGDM and GDM cases, respectively. This study was approved by the ethics committees of Qinhuangdao First Hospital and Qinhuangdao Maternal and Child Health Hospital.

The results of OGTT at 24–28 weeks of pregnancy were collected according to the GDM diagnostic criteria recommended by the International Association of Diabetes and Pregnancy Research Groups [5]: fasting blood glucose ≥ 5.1 mmol/L, lh blood glucose ≥ 10.0 mmol/L after taking sugar, and blood glucose > 8.5 mmol/L in the next 2 h; the diagnosis was confirmed when blood glucose levels met one or more criteria.

Exclusion criteria: ① Diabetes combined with pregnancy and overt diabetes during pregnancy; ② History of glucocorticoid application; ③ Hypertension; ④ Polycystic ovary syndrome; ⑤ Connective tissue diseases; ⑥ Liver and kidney diseases; ⑦ Other chronic diseases and pregnancy complications; ⑧ Recent history of acute infection; ⑨ Pregnant women with missing medical records.

### Methods

Determination of basic human body parameters: age, height, pre-pregnancy weight, mid-pregnancy weight, and other general conditions were recorded. The body mass index (BMI) was calculated using the formula: BMI = weight (kg)/height^2^ (m^2^).

Laboratory test data, including glycated hemoglobin (HbA1c), triglyceride (TG), total cholesterol (TC), high-density lipoprotein cholesterol (HDL-C), low-density lipoprotein cholesterol (LDL-C), uric acid (UA), blood urea nitrogen (BUN), creatinine (CREA), alanine aminotransferase (ALT), aspartate aminotransferase (AST), γ-glutamyltransferase (GGT), free triiodothyronine (FT3), free thyroxine (FT4), and thyroid-stimulating hormone (TSH), for 8–12 weeks were collected.

Statistical methods: All the statistical analyses were conducted using SPSS 25.0 software. The measurement data were expressed as mean ± standard deviation ($\overline{x}$±sd), and two-sample t-test was used for comparison between pregnant women in the GDM and NGDM group; the enumeration data were expressed as [cases (%)], and the χ^2^ test was used for comparison between groups. Significant indicators were further analyzed by logistic regression to analyze their correlation with GDM and then used in the prediction model. The discriminative power of the model was assessed using the receiver operating characteristic curve (ROC). The Hosmer–Lemeshow (HL) test was used to evaluate the calibration of the model. *P* < 0.05 indicated a statistically significant difference.

## Results

### The comparison results of the general and laboratory index of pregnant women in the modeling and verification cohorts

No significant difference was detected in height, weight gain in the second trimester, FT4, TSH, ALT, AST, BUN, and TC in the first trimester between the NGDM and GDM groups in the modeling cohort (*P* ≥ 0.05), while significant differences were noted in age, pre-pregnancy weight, mid-pregnancy weight, pre-pregnancy BMI, glycosylated hemoglobin, FT3, GGT, UA, CREA, TG, HDL-C, LDL-C, fasting plasma glucose (FPG), and blood glucose at 1 h and 2 h OGTT between the two groups (*P* < 0.05; Table 1).

**Table 1:** Comparison of the results of general pregnancy status and laboratory indicators in the modeling cohort

	NGDM	GDM	t	*P*
N	2975	1225	–	–
Age (years)	29.17 ± 4.01	30.38 ± 4.13	− 8.746	< 0.001
Height (cm)	162.85 ± 4.23	162.63 ± 4.69	1.420	0.156
Weight-1 (kg)	58.33 ± 8.82	62.30 ± 10.80	− 11.400	< 0.001
Weight-2 (kg)	64.30 ± 9.33	68.42 ± 10.83	− 11.644	< 0.001
BMI (kg/m^2^)	21.99 ± 3.19	23.54 ± 3.87	− 12.422	< 0.001
Weight-3 (kg)	5.97 ± 4.12	6.12 ± 3.76	− 1.110	0.267
HbA1c (%)	4.90 ± 0.29	5.16 ± 0.31	− 25.211	< 0.001
Glucose 0 h (mmol/L)	4.53 ± 0.33	5.20 ± 0.49	− 43.736	< 0.001
Glucose 1 h (mmol/L)	7.21 ± 1.22	9.30 ± 1.64	− 40.041	< 0.001
Glucose 2 h (mmol/L)	6.25 ± 0.98	7.85 ± 1.44	− 35.611	< 0.001
FT3 (pmol/L)	4.77 ± 0.74	4.86 ± 0.78	− 3.685	< 0.001
FT4 (pmol/L)	15.21 ± 3.28	15.03 ± 2.60	1.863	0.063
TSH (mIU/L)	1.64 ± 1.15	1.63 ± 1.09	0.222	0.824
ALT (U/L)	14.87 ± 13.21	15.65 ± 15.48	− 1.673	0.094
AST (U/L)	17.54 ± 7.47	17.65 ± 9.03	− 0.372	0.710
GGT (U/L)	12.80 ± 8.06	14.99 ± 10.43	− 6.576	< 0.001
UA (μmol/L)	201.41 ± 44.49	242.05 ± 63.95	− 20.579	< 0.001
BUN (mmol/L)	2.78 ± 0.98	2.81 ± 1.06	− 0.694	0.488
CREA (μmol/L)	44.31 ± 7.04	45.04 ± 7.89	− 2.820	0.005
TG (mmol/L)	1.53 ± 0.65	1.80 ± 0.76	− 11.097	< 0.001
TC (mmol/L)	4.62 ± 0.90	4.64 ± 0.85	− 0.534	0.593
HDL-C (mmol/L)	1.99 ± 0.49	1.81 ± 0.42	12.067	< 0.001
LDL-C (mmol/L)	1.88 ± 0.65	1.97 ± 0.69	− 4.137	< 0.001

GDM, gestational diabetes; NGDM, non-gestational diabetes; Weight-1, weight before pregnancy; Weight-2, weight in the second trimester; Weight-3, weight gain in the second trimester; BMI, body mass index; HbA1c, glycosylated hemoglobin, typeA1c; FT3, free triiodothyronine; FT4, free thyroxine; TSH, thyroid-stimulating hormone; ALT, alanine aminotransferase; AST, aspartate aminotransferase; GGT, gamma glutamyltransferase; UA, uric acid; BUN, blood urea nitrogen; CREA, creatinine; TG, triglyceride; TC, total cholesterol; HDL-C, high-density lipoprotein cholesterol; LDL-C, low-density lipoprotein cholesterol

In the verification cohort, no significant differences were detected in mid-gestational weight gain, FT4, TSH, AST, BUN, CREA, TC, and LDL-C of pregnant women in the NGDM and GDM groups (*P* ≥ 0.05). Conversely, age, height, pre-pregnancy weight, mid-pregnancy weight, pre-pregnancy BMI, glycosylated hemoglobin, FT3, ALT, GGT, UA, TG, HDL-C, FPG, OGTT1h blood glucose, and OGTT2h blood glucose differed significantly between the two groups (*P* < 0.05; Table 2).

**Table 2:** Comparative results of general conditions and laboratory indicators of pregnant women in the validation cohort

	NGT	GDM	t	*P*
n	1281	519	–	–
Age (years)	29.12 ± 4.07	30.40 ± 4.54	− 5.587	< 0.001
Height (cm)	162.80 ± 4.16	161.94 ± 4.21	3.992	< 0.001
Weight-1 (kg)	57.83 ± 8.24	61.20 ± 9.85	− 6.895	< 0.001
Weight-2 (kg)	63.93 ± 8.74	67.29 ± 9.79	− 6.801	< 0.001
BMI (kg/m^2^)	21.82 ± 3.02	23.33 ± 3.57	− 8.482	< 0.001
Weight-3 (kg)	6.11 ± 4.03	6.09 ± 3.84	0.077	0.939
HbA1c (%)	4.90 ± 0.29	5.14 ± 0.34	− 14.197	< 0.001
Glucose 0 h (mmol/L)	4.53 ± 0.33	5.21 ± 0.52	− 27.986	< 0.001
Glucose 1 h (mmol/L)	7.17 ± 1.25	9.40 ± 1.65	− 27.841	< 0.001
Glucose 2 h (mmol/L)	6.23 ± 0.98	7.94 ± 1.44	− 24.940	< 0.001
FT3 (pmol/L)	4.74 ± 0.78	4.93 ± 0.80	− 4.460	< 0.001
FT4 (pmol/L)	15.23 ± 4.34	15.34 ± 4.23	− 0.423	0.672
TSH (mIU/L)	1.57 ± 1.02	1.61 ± 1.01	− 0.807	0.420
ALT (U/L)	14.38 ± 12.34	16.26 ± 15.80	− 2.432	0.015
AST (U/L)	17.33 ± 7.74	17.70 ± 8.58	− 0.855	0.393
GGT (U/L)	12.73 ± 7.82	14.22 ± 8.50	− 3.447	0.001
UA (μmol/L)	203.09 ± 47.10	237.96 ± 63.72	− 11.281	< 0.001
BUN (mmol/L)	2.84 ± 1.07	2.87 ± 1.15	− 0.412	0.680
CREA (μmol/L)	44.54 ± 7.10	44.48 ± 7.43	− 0.156	0.876
TG (mmol/L)	1.47 ± 0.61	1.85 ± 0.85	− 9.027	< 0.001
TC (mmol/L)	4.65 ± 0.88	4.61 ± 0.88	0.718	0.473
HDL-C (mmol/L)	2.00 ± 0.48	1.87 ± 0.52	4.922	< 0.001
LDL-C (mmol/L)	1.88 ± 0.63	1.92 ± 0.69	− 1.123	0.262

GDM, gestational diabetes; NGDM, non-gestational diabetes; Weight-1, weight before pregnancy; Weight-2, weight in the second trimester; Weight-3, weight gain in the second trimester; BMI, body mass index; HbA1c, glycosylated hemoglobin, typeA1C; FT3, free triiodothyronine; FT4, free thyroxine; TSH, thyroid-stimulating hormone; ALT, alanine aminotransferase; AST, aspartate aminotransferase; GGT, gamma glutamyltransferase; UA, uric acid; BUN, blood urea nitrogen; CREA, creatinine; TG, triglyceride; TC, total cholesterol; HDL-C, high-density lipoprotein cholesterol; LDL-C, low-density lipoprotein cholesterol

### Multifactor logistic regression analysis results and prediction model were established

Multivariate logistic regression analysis was conducted on the significant variables (*P* < 0.05; Table 1). The results showed that age, BMI, glycosylated hemoglobin in the first trimester, UA, TG, and HDL-C were independent risk factors for GDM (*P* < 0.05; Table 3) and hence, were included in the logistic regression model; Y = 1/{1 + exp[− (− 18.373 + age × 0.065 + BMI × 0.030 + first-trimester HbA1c × 2.519 + UA × 0.014 + TG × 0.224-HDL-C × 0.635)]}.

**Table 3:** Results of the multivariate logistic regression analysis

	β	*P*	OR	95% CI
Age (years)	0.065	< 0.001	1.067	1.047–1.088
BMI (kg/m^2^)	0.030	0.011	1.031	1.007–1.055
HbA1c (%)	2.519	< 0.001	12.422	9.333–16.534
UA (μmol/L)	0.014	< 0.001	1.014	1.012–1.016
TG (mmol/L)	0.224	< 0.001	1.251	1.117–1.401
HDL-C (mmol/L)	− 0.635	< 0.001	0.530	0.445–0.631
constant	− 18.373	< 0.001	0.000	–

BMI, body mass index; HbA1c, glycosylated hemoglobin, typeA1C; FT3, free triiodothyronine; UA, uric acid; TG, triglyceride; HDL-C, high-density lipoprotein cholesterol

**Fig. 1:**
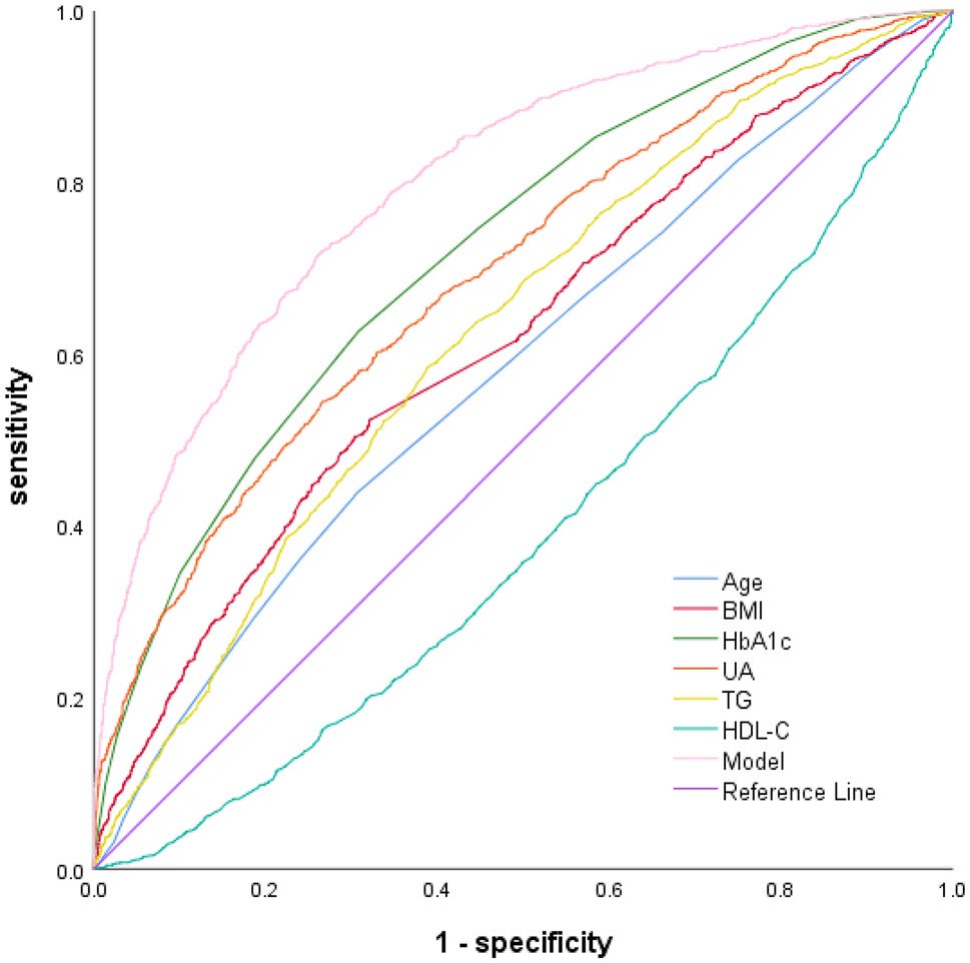
ROC curve of each indicator predicting the risk of GDM

The ability of the above indicators to predict the risk of GDM was analyzed according to the ROC curve, and the ability of the individual indicators to predict GDM was low (Table 4 and Fig. 1).

**Table 4:** The ability of each indicator to predict the risk of GDM

Index	Cutoff point	AUC	95% CI	Sensitivity (%)	Specificity (%)
Age (years)	30.5	0.583	0.563–0.602	43.8	69.3
BMI (kg/m^2^)	22.39	0.618	0.600–0.637	52.3	67.9
HbA1c (%)	5.05	0.722	0.705–0.738	62.6	69.2
UA (μmol/L)	226.55	0.693	0.676–0.711	54.4	73.4
TG (mmol/L)	1.53	0.626	0.608–0.644	58.4	61.1
Modeling cohort	0.28	0.803	0.788–0.817	72.0	73.5
Validation queue	0.28	0.782	0.759–0.806	68.6	73.8

BMI, body mass index; HbA1c, glycosylated hemoglobin, typeA1C; FT3, free triiodothyronine; UA, uric acid; TG, triglyceride; HDL-C, high-density lipoprotein cholesterol

Evaluation of the forecasting performance of the GDM forecast model discrimination test: The ROC curve with the sensitivity as the ordinate and 1-specificity as the abscissa. The area under the ROC curve (AUC) was 0.803 (*P* < 0.01), and the 95% confidence interval (CI) was 0.788–0.817. The Youden index was 0.455, the sensitivity was 72.0%, and the specificity was 73.5% (Table 4 and Fig. 1). Calibration test: The H–L test was selected to test the goodness-of-fit of the model. The values for the GDM risk prediction model were χ^2^ = 4.436, *P* = 0.816, and no significant difference was detected between the predicted and the actual observed values (*P* > 0.05).

**Fig. 2:**
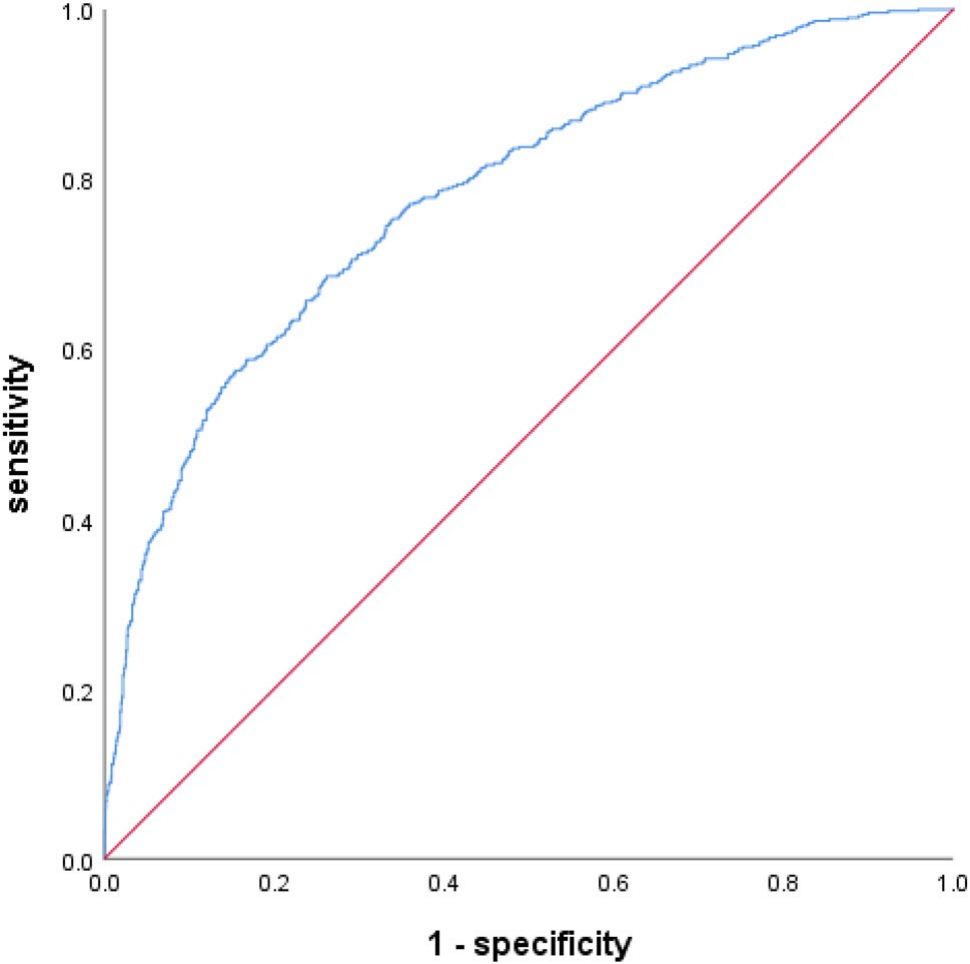
Area under the ROC curve of the validation queue

Validation cohort to evaluate the effectiveness of the GDM risk prediction model: The GDM prediction model was substituted in the first trimester into the validation cohort, and the ROC curve was constructed. The AUC was 0.782 (*P* < 0.01), and the 95% CI was 0.759–0.806. The Youden index was 0.424, the sensitivity was 68.6%, and the specificity was 73.8% (Table 4 and Fig. 2). The results of H–L test showed that the GDM risk prediction model in the first trimester was χ^2^ = 5.591, *P* = 0.693, and there was no significant difference between the predicted and the actual observed values (*P* > 0.05).

## Discussion

### GDM risk prediction model

GDM has gained increasing attention due to its hazardous outcomes and long-term adverse effects on mothers and offspring. Early detection and standardized management of GDM are essential to improve maternal and fetal outcomes [6]. Several scholars have established a GDM prediction model in early pregnancy to predict and intervene in the high-risk groups of GDM early, reducing the disease's occurrence and its complications and improving maternal and child outcomes. Sweeting et al. [7] included previous GDM medical history, family history of diabetes, age, race, parity, and BMI into the model, and the AUC was 0.88. When the model incorporated new maternal lipid markers, such as pregnancy-associated proteins, lipocalin-2, and triglycerides, the AUC was 0.91. The new model formed after the addition of new maternal lipid markers in the Sweeting model identified pregnant women at high risk of GDM more accurately than the old model, but lacks external data validation [8]. The prediction model of Teede et al. [9] includes previous GDM medical history, family history of diabetes, maternal age, pre-pregnancy BMI, and race. This model was simple and suitable for clinical application, but its predictive efficiency was low, with an AUC of 0.70. Wang et al. [10] applied four methods to establish a GDM risk prediction model in early pregnancy. The calculation of the scoring model was simple, but the AUC was 0.772, and the prediction performance was poor. The calculation formula of the logistic regression model was complicated but had a high accuracy; the AUC of training and validation sets was 0.799 and 0.834, respectively. Although the machine learning models had a high accuracy, achieving the same in clinical practice was challenging.

Some early pregnancy GDM prediction models have a good prediction performance but have not been widely used in clinical practice. The study of GDM prediction models in China started late, and a prediction model for GDM in the first trimester of pregnancy has not yet been established to provide a valuable preliminary screening tool for the early screening of pregnant women. This retrospective study analyzed the data of 6000 pregnant women. According to the clinical characteristics of pregnant women and laboratory results in the first trimester, a risk prediction model for GDM in the first trimester was established through logistic regression. The model finally included six predictors: age, pre-pregnancy BMI, HbA1c in the first trimester, UA, TG, and HDL-C. The AUC of the modeling cohort was 0.803 (95% CI: 0.788–0.817), with a sensitivity of 72% and a specificity of 73.5%. After substituting the equation into the validation cohort, the AUC was 0.782 (95% CI: 0.759–0.806), the sensitivity was 68.6%, and the specificity was 73.8%. The *P* values of the HL test for both the modeling and validation cohorts were > 0.05, indicating that the predictive model established in this study had a good fit.

### Correlation between clinical features and laboratory indicators in the first trimester and GDM

Some studies [11] have shown that the risk of GDM increases linearly with the age of pregnant women. The prevalence of GDM increases with maternal age [12]. Li et al. [13] found that advanced age, pre-pregnancy BMI overweight, and a history of diabetes in first-degree relatives are associated with an increased risk of GDM. In early pregnancy, age and pre-pregnancy BMI are independent risk factors for GDM, and the risk of GDM in overweight/obese women aged ≥ 35 years is 2.45 times that of normal women [14]. Our results were consistent with the above findings that age and pre-pregnancy BMI are independent risk factors for the occurrence and development of GDM. However, the ability of age and pre-pregnancy BMI to predict GDM was low, the AUC was 0.583 and 0.618, respectively, and the sensitivity and specificity were low.

In early pregnancy, high concentrations of TSH and FT3 and lower concentrations of FT4 were associated with an increased risk of GDM; pregnant women with a high FT3/FT4 ratio are more likely to suffer from GDM than normal pregnant women [15, 16]. Moreover, positive anti-peroxidase antibody (TPOAb) was also associated with an increased risk of GDM [14]. A retrospective analysis of 626 subjects [17] showed that elevated UA levels in early pregnancy were positively associated with GDM risk. High UA at 13–18 weeks of gestation is a risk factor for GDM, and in pregnant women ≥ 35-years-old, serum UA has a stronger correlation with GDM [18]. Li et al. [19] showed that high UA levels during 16–18 weeks of gestation were positively and independently associated with an increased risk of GDM, and those in the highest quartile increased the risk by 55.7% compared to the lowest quartile. In the present study, no significant difference was detected in the levels of FT4 and TSH between the two groups of pregnant women. The levels of FT3 and UA in the GDM group were significantly higher than those in the non-GDM group. However, after adjusting age, pre-pregnancy BMI, HbA1c, TG, HDL-C, and other factors in the first trimester, no correlation was established between FT3 level and GDM, while UA level was correlated with GDM and was an independent risk factor for GDM. When the UA in the first trimester was > 226.55 μmol/L, the possibility of pregnant women suffering from GDM was high, and the AUC was 0.693, which had a certain predictive ability.

HbA1c showed the average blood glucose level in the past 3 months. The HbA1c of pregnant women with GDM was significantly higher than that of pregnant women with normoglycemia. Women with higher HbA1c in the first trimester had a high risk of developing GDM [20]. Kattini et al. [21] found that the risk of GDM increased when the HbA1c level was > 5.7%, and all patients with GDM could be identified when the level was > 6.0%. Fasting blood glucose (FPG), OGTT1h blood glucose level, OGTT2h blood glucose level, and HbA1c level in early pregnancy are critical predictors of GDM, among which 1 h blood glucose level has the most significant predictive value [22]. Another study found that [23], the levels of TC and TG were significantly different between the GDM and the non-GDM groups. Cao et al. [24] speculated that compared to the normal pregnant subjects, TG, TC, low-density lipoprotein (LDL) and very low-density lipoprotein (VLDL) in GDM patients were significantly higher. Conversely, the high-density lipoprotein in the GDM group (HDL) concentration was low. In this study, HbA1c, TG, and HDL-C in the first trimester were independent risk factors for GDM, but HDL-C had no independent predictive effect on GDM. The AUCs of HbA1c and TG in the first trimester were 0.722 and 0.692, respectively, and the optimal cutoff points for predicting GDM were 5.05% and 1.53 mmol/L, respectively. Thus, focusing on the glucose and lipid metabolism levels of pregnant women in the first trimester of pregnancy to prevent the occurrence of GDM is imperative.

The occurrence of GDM can be predicted based on a single index; for example, HbA1c in the first trimester, but its sensitivity and specificity are low. However, whether it could predict the occurrence of GDM alone needs to be investigated further. Moreover, the current study found that compared to individual indicators, the risk prediction model established by combining age, pre-pregnancy BMI, and laboratory indicators in the first trimester can increase the AUC from 0.583–0.722 to 0.803; also, the sensitivity and specificity have been improved.

This study mainly used factors that were easy to obtain, identify, and intervene, such as the results of early pregnancy checkups of pregnant women, as predictors, and incorporated thyroid function indicators and UA in the first trimester into the GDM risk prediction model to provide a basis for the identification of high-risk groups for GDM. Nevertheless, the present study has several deficiencies. Herein, only the pregnant women of Qinhuangdao City were included, which could not be used to infer the situation in other regions. The fitting degree of the predictive model was good, but the AUCs of the modeling cohort and the validation cohort were 0.803 (95% CI: 0.788–0.817) and 0.782 (95% CI: 0.759–0.806), respectively, and the predictive power was moderate. Also, the prediction model had not been verified externally, and needs further extrapolation.
